# Illness Caused by Filth in Milk

**Published:** 1882-07-20

**Authors:** 


### ILLNESS CAUSED BY FILTH IN MILK.

Dr. C. A. Cameron, Chief Medical Officer of Health, for Dublin, reports, in the Dublin Journal of Medical Science, the following interesting facts concerning a hitherto scarcely noted cause of milk impurity: A specimen of milk was submitted to him for examination, which was believed to contain a taxic substance of some kind, for the following reasons: Three children who were in the habit of drinking largely uncooked milk, were taken sick. They presented furred tongues, and gastric symptoms, such as are usually present in the earlier stages of enteric fever. Temperature 104-5° F. The house was new, in a healthy location, and no bad odors had been noticed. The sanitary arrangements were in goood order. The children had been healthy two days before. A general examination of the mild in stock was made; It presented a peculiar appearance, the cream which had risen to the surface having a deep brown color. A short time before the children's illness a similar brown stratum had been observed on the milk. Generally the milk presented no peculiar features. The composition was found to be:

| | |
|---|---|
| Water | 87.10 per cent. |
| Fats | 3.56 per cent. |
| Other solids | 8.34 per cent. |
| Total | 100.00. |

It was, therefore, milk of good quality; but a microscopical examination of the cream taken from this milk revealed the presence of cow's hairs, minute particles of straw, and *debris* of organic matter in great abundance. There were numerous nomads, vibrios and bacteroid bodies. The odor of the cream was slightly but distinctly unpleasant. A subsequent visit to the dairyman was made. He at firse loudly protested that he sold only *pure* milk. The cows were examined and found healthy; no cause whatever could be discovered for the impurities. Finally, on pressing the milk vender for an explanation, he stated that the cows were milked early in the morning, by his nephew, who had no light with him, and omitted to wash the teats of the cows before allowing their milk to flow into the milk pails. As some of the cows had lain down all night in such a way that their udders were in contact with the manure in or close to the channel courses, their teats were covered with filth. (A word to the wise is sufficient; this report demonstrates how easily good, pure milk can be rendered poisonous, and the remedy suggests itself at once to every intelligent man.—ED.)—*Med. and Surg. Rep.*

